# Cognitive reserve, cortisol, and Alzheimer's disease biomarkers: A memory clinic study

**DOI:** 10.1002/alz.13866

**Published:** 2024-06-04

**Authors:** Manasa Shanta Yerramalla, Alexander Darin‐Mattsson, Chinedu T Udeh‐Momoh, Jasper Holleman, Ingemar Kåreholt, Malin Aspö, Göran Hagman, Miia Kivipelto, Alina Solomon, Anna Marseglia, Shireen Sindi

**Affiliations:** ^1^ Division of Clinical Geriatrics, Department of Neurobiology, Care Sciences, and Society Karolinska Institutet Stockholm Sweden; ^2^ Aging Research Center Karolinska Institutet and Stockholm University Stockholm Sweden; ^3^ Brain and Mind Institute The Aga Khan University Nairobi Kenya; ^4^ Department of Epidemiology and Prevention Wake Forest University Winston‐Salem North Carolina USA; ^5^ Institute of Gerontology School of Health and Welfare Jönköping University Jönköping Sweden; ^6^ Theme Inflammation and Aging Karolinska University Hospital Stockholm Sweden; ^7^ Ageing Epidemiology Research Unit (AGE), School of Public Health, Faculty of Medicine Imperial College London London UK; ^8^ Institute of Public Health and Clinical Nutrition University of Eastern Finland Kuopio Finland; ^9^ Institute of Clinical Medicine, Neurology University of Eastern Finland Kuopio Finland

**Keywords:** amyloid beta, cerebrospinal fluid biomarkers, cognitive performance, cognitive reserve, memory clinic, perceived stress, phosphorylated tau, salivary cortisol, total tau

## Abstract

**INTRODUCTION:**

Cognitive reserve might mitigate the risk of Alzheimer's dementia among memory clinic patients. No study has examined the potential modifying role of stress on this relation.

**METHODS:**

We examined cross‐sectional associations of the cognitive reserve index (CRI; education, occupational complexity, physical and leisure activities, and social health) with cognitive performance and AD‐related biomarkers among 113 memory clinic patients. The longitudinal association between CRI and cognition over a 3‐year follow‐up was assessed. We examined whether associations were influenced by perceived stress and five measures of diurnal salivary cortisol.

**RESULTS:**

Higher CRI scores were associated with better cognition. Adjusting for cortisol measures reduced the beneficial association of CRI on cognition. A higher CRI score was associated with better working memory in individuals with higher (favorable) cortisol AM/PM ratio, but not among individuals with low cortisol AM/PM ratio. No association was found between CRI and AD‐related biomarkers.

**DISCUSSION:**

Physiological stress reduces the neurocognitive benefits of cognitive reserve among memory clinic patients.

**Highlights:**

- Physiological stress may reduce the neurocognitive benefits accrued from cognitively stimulating and enriching life experiences (cognitive reserve [CR]) in memory clinic patients.
- Cortisol awakening response modified the relation between CR and P‐tau_181_, a marker of Alzheimer's disease (AD).
- Effective stress management techniques for AD and related dementia prevention are warranted.

## 1 BACKGROUND

Amyloid beta 1‐42 (Aβ_42_) senile plaques and tau neurofibrillary tangles are hallmarks of Alzheimer's disease (AD) pathology.^1^ The clinical manifestation of AD varies among individuals despite similar neuropathological burden.^2^ Cognitive reserve (CR) suggests that individual differences in the cognitive processes underlying task performance allow some people to cope better than others with accumulating neuropathological changes.^3^ Cognitively stimulating and enriching life experiences and behaviors, such as early‐life cognitive enrichment, high educational attainment, complex jobs, and sustained physical, mental, and social engagement, ^4^, ^5^, ^6^, ^7^, ^8^ help build CR, potentially explaining why age‐related brain changes do not always lead to cognitive impairment and dementia. Among individuals with mild cognitive impairment (MCI), studies have highlighted the potential protective role of CR proxies such as higher educational attainment, work activity, and leisure time against cognitive decline and subsequent progression to dementia.^9^ But most studies have used a single‐domain and early‐life proxy such as educational level to represent CR, while few have accounted for late‐life leisure activities, and none have considered the role of late‐life social health as a proxy marker,^9^ even though it positively contributes to cognitive capabilities.^5^, ^10^ Moreover, given that individuals with subjective cognitive decline (SCD) and MCI are at high risk of progression to dementia,^11^, ^12^ the modifying role of chronic health‐related risk factors (eg, cardiometabolic conditions or depression) in the association between CR, AD‐related pathology, and cognition among memory clinic patients’ also needs to be understood.

Among emerging modifiable risk factors for the development of MCI toward dementia, stress has gained prominence, including psychological (ie, emotional/mental strain in response to challenging or threatening situations, threats to one's mental/emotional integrity) and physiological stress (ie, the body's physical response to stressors, activating the “fight or flight” response, and changes in hormonal levels and other bodily reactions).^13^ Prior research employed various stress measures, from subjective (eg, perceived stress) to biological (eg, salivary cortisol), both interconnected yet distinct in nature.^14^, ^15^ High levels of perceived chronic stress have been associated with faster cognitive decline among individuals with MCI and subsequent risk of progression from MCI to dementia.^16^, ^17^, ^18^ Psychological stress has also been shown to be markedly elevated in individuals with MCI compared to non‐amnestic cognitively healthy adults.^13^, ^19^ Systematic reviews have found elevated levels of cortisol to be associated with impaired cognition and increased risk of AD and a potential contributor to AD pathology.^20^, ^21^ Among MCI groups, high salivary cortisol levels but not psychological stress were found to be associated with poorer cognitive function cross‐sectionally.^13^ Additionally, high or persistent levels of stress have been related to spending less amount of time in leisure activities,^22^ frequent retractions from social interactions,^23^ and impairing one's ability to be physically active.^24^ It is unclear, however, how stress relates to the link between CR and cognition as well as AD‐related biomarkers.

Individuals in the prodromal stage of AD can potentially reverse their condition, and this stage is considered a “window” during which it is still possible to intervene to avoid or delay the onset of dementia.^25^ In memory clinic participants, CR is in part operationalized through multidomain interventions consisting of cognitively stimulating activities and physical activity, for example, which could potentially improve cognitive outcomes.^26^ Consequently, to determine whether stress needs to be viewed as a risk factor preventing the full realization of the benefits of CR, it is necessary to examine whether the interaction between CR and stress may increase the likelihood of cognitive impairment and AD‐related pathology among memory clinic participants.

To our knowledge, no study has examined multiple biological indicators of physiological stress in relation to CR‐cognition and CR‐AD biomarker association in memory clinic patients. Our study aimed to assess cross‐sectional associations between CR, cognitive performance, and AD biomarkers in patients from a Swedish memory clinic. We examined whether the protective role of CR might be dependent on stress levels. We also examined the association between CR and cognitive trajectories over 3 years of follow‐up. The potential modifying roles of age and biological sex at birth were also addressed.

## 2 METHODS

### 2.1 Study population

**FIGURE 1 alz13866-fig-0001:**
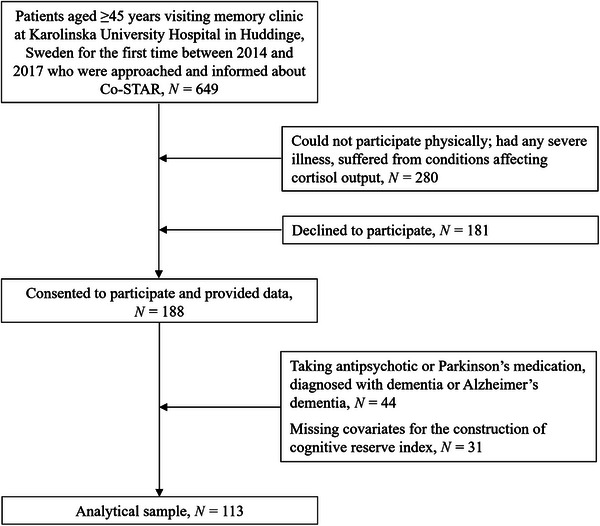
Participant flow chart.

This study is based on the Cortisol and Stress in Alzheimer's disease (Co‐STAR) cohort study investigating the role of stress and lifestyle factors among patients referred to the memory clinic at the Karolinska University Hospital, Huddinge (Sweden). Patients aged 45 or more years who had attended their first visit to the memory clinic between the years 2014 and 2017 (*N* = 649) were invited to participate. Of the 649 patients who were approached and informed about Co‐STAR, 280 were excluded as they were physically incapable of participating, had severe sensory impairments (eg, auditory, cognitive, or visual), or had conditions that affected their hypothalamic‐pituitary‐adrenal (HPA‐axis) activity (eg, Cushing's disease), 181 did not consent to participate, and 188 consented to participate and provided data (Figure 1). Excluding individuals diagnosed with dementia or Alzheimer's dementia to reduce reverse causation, those taking antipsychotics or medication for Parkinson's that might impact their cognition and/or ability to engage in activities, or those missing covariates led to an analytical sample of 113 participants. Compared with the participants included (*n* = 113) in the analyses, participants not included (*n* = 75) did not differ by age, sex, and education (data not shown). Participants diagnosed with SCD or MCI at baseline were invited for subsequent follow‐up examination. After an average follow‐up of 32 months, 68 of the 123 participants invited completed follow‐up assessments between February 2018 and May 2019. Of the 68 participants who completed follow‐up assessments, analyses were conducted for 45 and 43 participants with data on memory and processing speed, respectively at a mean follow‐up of 2.71 (standard deviation [SD] = 0.73) years. Research ethics approval for Co‐STAR study was obtained from the Regional Ethical Review Board (Stockholm, reference number: 2014/524‐31/1). Only participants whose informed written consent was obtained were included in the data collection.

**RESEARCH IN CONTEXT**

1. **Systematic review**: The authors reviewed the literature using traditional sources (eg, PubMed). No prior study examined the role of multiple biological indicators of physiological stress in relation to cognitive reserve (CR)‐cognition and CR‐Alzheimer's disease (AD) biomarker association among memory clinic patients.
2. **Interpretation**: In a cross‐sectional sample of memory clinic patients, salivary diurnal cortisol measures appeared to reduce the neurocognitive benefits accumulated via resilience‐enhancing experience and late‐life lifestyle. A beneficial relation between CR and working memory was apparent among patients with high/favorable cortisol AM/PM ratio but not in those with low cortisol AM/PM ratio. CR was not related to AD biomarkers, although there was an indication of a potential modifying role of cortisol awakening ratio in the relation between CR and tau pathology. No association between CR and cognition over a 3‐year follow‐up was observed.
3. **Future directions**: Future studies should examine the potential effectiveness of stress management techniques in AD prevention.

### 2.2 Data collection and clinical assessments

As part of the standard assessment protocol at the Karolinska University Hospital memory clinic, eligible participants had to undergo routine clinical assessments. This included meeting with neuropsychologists for a comprehensive neuropsychological test battery, complete physical and neurological examinations and undergoing brain imaging (mainly magnetic resonance imagining [MRI]), and collection[Fig alz13866-fig-0001] of blood and cerebrospinal fluid (CSF) samples. Co‐STAR participants were additionally provided with a home cortisol sampling kit and several questionnaires. Participants were diagnosed with dementia based on a consensus meeting using the dementia diagnostic criteria of the International Classification of Diseases 10th revision (ICD‐10).^27^ A diagnosis of MCI was given in accordance with the criteria provided by Winblad and colleagues,^28^ which includes subjective cognitive complaints not typical for age, daily functional activities being essentially preserved, evidence of cognitive decline measured by objective cognitive testing, and not fulfilling the diagnostic criterion for dementia. If participants did not fulfill the diagnostic criterion for either dementia or MCI but reported self‐perceived decline in cognitive abilities, they were classified as having SCD.

### 2.3 Ascertainment of outcomes

#### 2.3.1 Cognitive performance

All participants underwent extensive cognitive testing. Raw test scores were *z*‐standardized and averaged to create composite scores for four cognitive domains (memory, processing speed, working memory, and perceptual reasoning) and global cognition. The episodic/short‐term memory score (hereafter referred to as memory) was based on four tests, namely, the Rey Auditory Verbal Learning Test (delayed recall),^29^ the Rey‐Osterrieth Complex Figure test (immediate recall),^30^ the Wechsler Adult Intelligence Scale (WAIS) Digit Symbol Substitution Test (immediate recall),^31^ and the Hagman test, which was developed and is utilized at the Karolinska University Hospital memory clinic, Huddinge, to assess visual memory (manuscript under preparation). Processing speed was assessed with the WAIS Digit Symbol Substitution Test.^32^ Working memory was computed using two tests: WAIS Digit Span and WAIS Arithmetic. Perceptual reasoning was assessed using an index created from WAIS Block Design and WAIS Matrix Reseasoning.^31^ A global cognitive score, composed of four subtests (Block Design, Similarities, Matrix Reasoning, and Information) of the Wechsler Abbreviated Scale of Intelligence (WASI) test, was obtained.^33^, ^34^ The two subtests of the WAIS, namely, Similarities and Information, were related to verbal cognition, while the other two (Block design and Matrix Reasoning) are related to non‐verbal cognition.^35^ Only two domains of cognitive performance, namely, memory and processing speed, were available at follow‐up, thus considered for longitudinal data analysis.

Given that the composite *z*‐score in each domain did not follow a normal distribution and logarithm transformation did not improve the distribution, these scores were dichotomized with a cut‐off of zero, which represented the mean cognition scores in a cognitively healthy sample of participants. Based on a reference of cognitively healthy sample of 24 older Swedish adults (men = 12; women = 12), unadjusted standardized *z*‐scores for all cognitive tests were calculated. The sample of healthy volunteers (male = 63.2 years, range = 47 to 75 years) did not differ in terms of age with the study sample (male = 62.5 years, range = 47 to 82 years), although they had marginally higher levels of education (healthy reference sample: male = 17.0 years, SD = 3.1; study sample: male = 14.2 years, SD = 3.2). Compared to the healthy reference sample, participants in the study sample were dichotomized as either having below (hereafter referred to as impaired cognitive function) or above (not having impaired cognitive function) average cognitive function. When the composite *z*‐score for cognitive performance was >0, the corresponding dichotomized score was classified as one, indicating that cognitive performance was above average level in the healthy reference and is the favorable group.

#### 2.3.2 CSF biomarkers for AD

Three CSF biomarkers were included in the study: Aβ_42_, phosphorylated tau 181 (p‐tau_181_), and total tau (t‐tau). The CSF samples were obtained through lumbar puncture using polypropylene tubes, gently mixed to avoid gradient effects, centrifuged for 10 min at 2000×g, and subsequently kept at −80°C until biochemical analysis. Aβ_42,_ t‐tau, and p‐tau_181_ were measured by means of sandwich enzyme‐linked immunosorbent assay, previously described in more detail for Aβ_42_ ^36^ and for p‐tau_181_ and t‐tau.^37^ Given their non‐normal distribution, t‐tau and p‐tau_181_ underwent logarithmic transformation to reduce skewness.

### 2.4 Assessment of cognitive reserve

An indicator of CR was computed by combining four candidate CR proxies: lifetime education, occupational complexity, late‐life social health index, and late‐life leisure activities index. ^5^, ^6^, ^8^ The details are as follows:

***Education***: Years of education were assessed by a questionnaire. The average number of years of education was 14 (SD = 3.3 years; range: 7 to 26 years).

***Substantive occupational complexity***: As a measure of occupational complexity, the substantive complexity of the participants’ longest‐held occupation was measured. This measure was developed by Roos and Treiman^38^ and is based on the U.S. Dictionary of Occupational Titles using the US Census of 1970.^38^ Substantive complexity comprises eight characteristics that is, general education development, complexity of work with data, intellectual aptitude, numerical aptitude, verbal aptitude, temperament for repetitive and continuous processes, and abstract interest in jobs, which were standardized and summed to create an interpretable scale ranging from 0 to 10, with higher scores indicative of more complex occupation.^38^, ^39^ Occupations of the participants were coded with the Nordic Occupational Classification, a grid that matched occupational categories in the 1970 US census to occupations from the 980 Swedish census to create substantive complexity scores for Swedish occupations.^40^ See Darin–Mattsson et al.^41^ and Andel et al.^40^ for a more detailed description of occupational complexity and the matching procedure, respectively. Two independent evaluators validated these indices for both the translation and coding of occupation of participants. The resulting substantive complexity score for our sample population ranged from 1.7 to 10 on a continuous scale, with a mean [SD] of 6.47 [2.10].

***Social health index (late life)***: A composite score was created to represent social health in late life based on questionnaire data about social networks and accounting for the dimensions of the network size and the quality of the support received.^5^ This consisted of two main components (see supplementary method for questionnaire items): (1) social connection (marital status, frequency of direct or remote contact with family, friends, and relatives and network size); (2) social support (12 questions pertaining to perceived satisfaction with aforementioned contacts in providing emotional support and instrumental aid). Raw scores on the four items under social connection and the 12 items under social support were standardized into *z*‐scores separately within their specific components to generate a *social connection index* and a *social support index*. The two standardized indices were averaged to generate an overall social health index (SHI) (Figure S1). There was moderate correlation of 0.46 between the two indices. The resulting variable, SHI, was a continuous score ranging from −1.66 to 1.32, where a higher score was indicative of having better social health.

***Leisure activities index (late life)***: Late‐life engagement in 28 activities was assessed across five questions where participants were instructed to indicate the ones they participated in and specify the frequency of engagement in the preceding year (see supplementary method for questionnaire items). These activities were categorized into three domains of leisure activities, namely, social (11 activities), mental (10 activities), and physical (seven items). Frequency of participation was assessed based on a five‐item response scale, that is, never, rarely, two to three times per month, several times per week, and every day, which were coded from 0 to 4, respectively. The richness of engagement within each domain was derived by weighting the frequency of activity participation by assigning a value of 1 for every week, 0.66 for every month, 0.33 for less frequently, and 0 for never, and then the scores were summed within their respective domains. This was done as individuals might have the same number of activities, but the level of engagement could be different, and thus a weighted sum was preferred over simply summing the number of activity scores, as was previously done.^42^, ^43^ The richness of engagement within each domain was then categorized into three levels (low, moderate, and high) and coded between 0 and 2, respectively. It has been shown that reductions in dementia risk are similar across all three leisure components and that the largest risk reduction is achieved with rich engagement in all domains.^44^ Given this, previous studies^42^, ^43^ generated a composite leisure score. Hence, a global measure of leisure richness was constructed by summing the scores of social, mental, and physical components (Figure S2), resulting in a continuous variable referred to as a *leisure activity index* ranging from 0 to 6, where a higher score denotes greater engagement in leisure activities.

All four of the aforementioned measures of cognitively, socially, and physically stimulating activities were standardized, and their *z*‐scores were averaged to derive a continuous indicator, namely, the cognitive reserve index (CRI). This normally distributed indicator with a mean of zero ranged from −1.68 to 1.48, with higher values reflecting greater CR.

### 2.5 Stress measures

The manner in which stress has mainly been assessed in research can be broadly classified into three different perspectives: environmental, psychological, and biological.^45^ This study focuses on psychological and biological perspectives. Psychological stress, assessing subjective stress appraisal, and affective reaction were measured using the Perceived Stress Scale (PSS).^46^ The PSS questionnaire was designed to measure “the degree to which individuals appraise situations in their lives as stressful”.^46^ The PSS items evaluate the degree to which individuals believe their life has been unpredictable, uncontrollable, and overloaded during the last month.^46^, ^47^ These items are general in nature and do not focus on specific events or experiences.^47^ The 10‐item PSS (PSS‐10) was administered as part of the self‐reported questionnaire, which is short and easy to use with acceptable psychometric properties to measure perceives stress, in both research and practice.^47^

Salivary cortisol, which is frequently employed in stress research^15^ because it reflects physiologically active free cortisol,^48^ was used to measure the diurnal cortisol pattern. Participants were instructed not to brush or floss their teeth, eat, drink, or smoke before the samples were collected. Saliva sample collection was done by participants using an at‐home sample collection kit using a passive drool technique. Participants were asked to collect a total of six saliva samples on each of the two non‐successive weekdays to account for day‐to‐day variability in cortisol levels. The timing of the sample collection was on waking (time point [t1]), 30 min after waking (t2), 60 min after waking (t3), at 2:00 p.m. (t4), at 4:00 p.m. (t5), and at bedtime (t6). Participants recorded the precise time each saliva sample was collected and were asked to freeze the samples until they could be sent to the memory clinic in a protected container. Following that, the saliva samples were sent to Dresden LabService GmbH (Dresden, Germany), where they were kept at −20°C pending analysis. Prior to being analyzed, samples were thawed and centrifuged at 3000 rpm for 5 min to produce a low viscosity. Salivary cortisol levels were measured using extremely sensitive chemiluminescence immunoassay (IBL International Hamburg, Germany), with intra‐ and interassay coefficients of variance of less than 8%.

Anomalous salivary profiles were excluded, consistent with previous studies:^49^, ^50^ these included morning measurements of t1 and t2 taken more than 15 min over or under their intended time of measurement and cortisol concentration more than three SD from the mean. Measurements were averaged over the 2 days of data collection. Three cortisol measures extensively used in stress research^50^ were derived: (1) the cortisol awakening response, calculated as the “area under the curve with respect to increase” from t1 to t2^51^; (2) total daily cortisol output, calculated as the “area under the curve with respect to ground”.^51^ for t1, t2, t4, t5, and t6; and (3) diurnal variation termed as cortisol AM/PM ratio, calculated by the awakening cortisol levels (t1) divided by the sample taken at bedtime (t6). The cortisol awakening response and total daily cortisol output were computed without t3 since a considerable proportion of participants had erroneous t3 data for both assessment days. In this study, five salivary cortisol measures were used for analysis: awakening cortisol (t1) levels, bedtime cortisol (t2) levels, the cortisol awakening response (CAR), total daily cortisol output, and the cortisol AM/PM ratio. All measures except for the cortisol awakening response were non‐normally distributed and thus underwent logarithmic transformation to increase their normality.

### 2.6 Statistical analysis

Descriptive characteristics of the study participants as a function of CRI were examined using Student's *t*‐test, chi‐square test and fisher's exact test whenever appropriate. Given no significant differences between use of medication (cardiometabolic disorder [antihypertensive, lipid‐lowering, and diabetes], antidepressants, and sleep disturbances), smoking status, and body mass index (BMI) across the CRI (Table 1), and owing to sample size constraints, the main model adjustment was made for age and sex (Model 1).

**TABLE 1:** Characteristics of study participants by cognitive reserve index.

		Cognitive reserve index		
	*N*	Low (*n* = 57)	High (*n* = 56)	*p*
**Demographics and lifestyle**				
Age (years), M (SD)	113	61.81 (8.03)	62.22 (7.38)	0.781
Education (years), M (SD)	113	12.71 (2.89)	15.91 (2.88)	***<.001***
Women	113	30 (52.63)	33 (58.93)	0.500
Current or ex‐smokers	108	36 (66.67)	31 (57.41)	0.321
Body mass index, M (SD)	51	27.69 (3.82)	27.00 (3.33)	0.492
**Use of medications**				
Antihypertensive	112	20 (35.09)	16 (29.09)	0.497
Lipid lowering	112	7 (12.28)	8 (14.55)	0.725
Diabetes	112	8 (14.04)	6 (10.91)	0.617
Total cardiometabolic medications, M (SD)	112	0.61 (0.77)	0.55 (0.79)	0.643
Antidepressants	112	18 (31.58)	14 (25.45)	0.473
Sleep disturbances	112	10 (17.54)	7 (12.73)	0.478
**Cognition, *z*‐score M (SD)**				
Global cognition	91	−0.42 (1.02)	0.39 (0.81)	***<.001***
Memory	98	−0.13 (0.95)	0.12 (1.04)	0.204
Processing speed	92	−0.15 (0.92)	0.50 (0.96)	***0.001***
Working memory	78	−0.26 (0.95)	0.23 (1.00)	***0.030***
Perceptual reasoning	93	−0.31 (1.00)	0.29 (0.91)	***0.003***
**Stress measures**				
Perceived Stress Scale, (_/40) M (SD)	109	19.40 (6.81)	16.07 (7.06)	***0.014***
**Cortisol measures**				
Awakening cortisol (t1), nmol/L	102	9.17 (5.76)	9.04 (6.11)	0.910
Bedtime cortisol (t6), nmol/L	102	3.09 (9.12)	2.64 (4.74)	0.751
Cortisol awakening response	101	0.79 (1.33)	0.53 (1.15)	0.286
Daily cortisol output	101	87.59 (63.14)	82.63 (77.38)	0.725
Cortisol AM/PM ratio (t1/t6)	102	7.01 (5.19)	9.91 (8.76)	***0.045***
**AD‐related CSF biomarkers, M (SD)**				
Aβ_42_, ng/L	93	816.94 (205.64)	809.07 (227.43)	0.861
T‐tau, ng/L	93	5.63 (0.45)	5.66 (0.48)	0.774
P‐tau_181_, ng/L	93	3.69 (0.37)	3.75 (0.39)	0.488

*Note*: Values are *n* (column%) unless otherwise stated. Bold Italicized is *p* value significant at < .05. A higher score on the cognitive reserve index (CRI) is indicative of better cognitive reserve, that is, an individual has better ability to cope with AD‐related pathological changes in the brain. The CRI was categorized into low and high groups based on the median cut‐off value. The low CRI ranged between −1.68 and 0.016 (M [SD] = −0.53 [0.42]), while the high CRI ranged from 0.017 to 1.48 (M [SD] = 0.54 [0.38]). Total cardiometabolic medications include the count of the following medications: antihypertensives, lipid‐lowering, and antidiabetic agents.

Abbreviations: AD, Alzheimer's disease; CSF, cerebrospinal fluid; M, mean; SD, standard deviation.

Logistic regression models were used to estimate the cross‐sectional association of CRI with global and domain‐specific cognitive performance, adjusted for Model 1 (age and sex). Model 1 was further adjusted for each of the six stress variables (PSS, awakening cortisol, bedtime cortisol, CAR, daily cortisol output, and cortisolAM/PM ratio) in separate regression models for each of the cognitive outcome. Since there were 30 cross‐sectional multiple regression analyses, *p* values for the stress coefficients were adjusted using the Simes–Benjamini–Hochberg false discovery rate (FDR) method,^52^ and both unadjusted and FDR‐adjusted *p* values have been reported. The FDR‐adjusted *p* value (ie, *q* value) threshold was 0.05. As sleep is regarded as an important factor in the stress‐health models^53^; we additionally adjusted Model 1 with use of sleep medication to ensure the robustness of our findings.

The associations of baseline CRI with memory and processing speed at follow‐up were modeled using logistic regression, with adjustments made for Model 1 and domain‐specific cognitive performance at baseline. Linear regression analyses were conducted to assess the association between CRI and AD‐related CSF biomarkers. Interactions were assessed between CRI and age (continuous), sex (male versus female), and each of the six measures of stress (continuous). When interactions were significant at *p *< 0.10,^54^ stratified analyses were conducted. Data analyses were undertaken using Stata SE, version 17.0 (StataCorp, College Station, TX) with a two‐sided *p* < 0.05 considered statistically significant. The Stata qqvalue package was used to obtain *p* values adjusted for multiple comparisons.^52^

## 3 RESULTS

### 3.1 Participant characteristics

The characteristics of participants as a function of CRI are shown in Table 1. The CRI score was categorized into low (unfavorable) and high (favorable) groups based on median cut‐off value, with a higher score being indicative of better CR. Participants with a low CRI score were more likely to be less educated, have poorer cognitive performance in the domains, have higher perceived stress scores, and have lower cortisol AM/PM ratios than individuals with high CRI scores (Table 1). The correlations between the five cortisol measures ranged from 0.03 (between bedtime cortisol levels and cortisol awakening response) to 0.83 (between bedtime cortisol and cortisol AM/PM ratio levels) in absolute terms (Table S1). The PSS‐10 had a negligible or low degree of correlation with salivary cortisol measures (*r* = 0.01 to 0.21 in absolute terms, Table S1).

### 3.2 Associations between CRI and cognitive performance

The cross‐sectional associations of CRI with cognitive domains are shown in Table 2, Model 1. In logistic regression models adjusted for age and sex, a higher CRI score was associated with increased odds of better global cognition (odds ratio [OR]: 5.22, 95% confidence interval [CI]: 1.90 to 14.36), processing speed (OR: 2.64, 95% CI: 1.14 to 6.13), working memory (OR: 3.21, 95% CI: 1.21 to 8.55), and perceptual reasoning (OR: 3.86, 95% CI: 1.63 to 9.17). There was no significant association between CRI and memory at baseline. During follow‐up (mean = 2.71 years, range = 1.25 to 4.26 years), baseline CRI was not associated with change in memory or processing speed (Table 3). When sensitivity analyses were performed by removing participants taking antidepressants (*n* = 32), only the association between CRI with global cognition (*p* = 0.006) and perceptual reasoning (*p* = 0.007) remained significant (data not shown).

**TABLE 2:** Association of cognitive reserve index with cognitive performance among participants from memory clinic with subjective cognitive impairment or mild cognitive impairment.

	Global cognition (*N* = 91)		Memory (*N* = 98)		Processing speed (*N* = 92)		Working memory (*N* = 78)		Perceptual reasoning (*N* = 93)	
CRI score	OR (95% CI)	*p*	OR (95% CI)	*p*	OR (95% CI)	*p*	OR (95% CI)	*p*	OR (95% CI)	*p*
**Model 1**	5.22 (1.90 to 14.36)	***0.001***	1.53 (0.72 to 3.26)	0.265	2.64 (1.14 to 6.13)	***0.024***	3.21 (1.21 to 8.55)	***0.019***	3.86 (1.63 to 9.17)	***0.002***
**Adjustment for stress measures**										
**Perceived stress scale**	6.86 (2.11 to 22.32)	***0.001***	1.88 (0.82 to 4.32)	0.139	2.52 (1.02 to 6.28)	***0.046***	3.70 (1.27 to 10.78)	***0.016***	4.52 (1.72 to 11.89)	***0.002***
**Awakening cortisol**	5.27 (1.74 to 15.91)	***0.003***	1.69 (0.73 to 3.93)	0.221	2.34 (0.91 to 5.97)	0.077	2.72 (0.98 to 7.56)	0.055	2.96 (1.19 to 7.34)	***0.019***
**Bedtime cortisol**	4.81 (1.59 to 14.49)	***0.005***	1.60 (0.68 to 3.74)	0.278	2.50 (0.98 to 6.35)	0.055	2.58 (0.93 to 7.14)	0.068	2.68 (1.07 to 6.68)	***0.035***
**CAR**	5.09 (1.68 to 15.40)	***0.004***	1.95 (0.81 to 4.71)	0.139	2.46 (0.97 to 6.24)	0.059	2.70 (0.98 to 7.45)	0.056	2.84 (1.15 to 7.02)	***0.024***
**Daily cortisol output**	5.07 (1.68 to 15.33)	***0.004***	1.72 (0.74 to 4.01)	0.207	2.44 (0.97 to 6.14)	0.058	2.71 (0.98 to 7.53)	0.055	2.82 (1.14 to 6.99)	***0.025***
**Cortisol AM/PM ratio**	4.49 (1.46 to 13.78)	***0.009***	1.57 (0.66 to 3.73)	0.307	2.29 (0.88 to 5.98)	0.091	2.80 (0.98 to 7.99)	0.054	2.60 (1.03 to 6.57)	***0.043***

*Note*: Bold italicized is *p* value significant at < 0.05.

Model 1 is adjusted for age and sex. Adjustment for stress is for six different stress measures separate from Model 1.

Abbreviations: CAR, cortisol awakening response; CI, confidence interval; cortisol AM/PM ratio, awakening cortisol/bedtime cortisol; CRI, cognitive reserve index; OR, odds ratio.

**TABLE 3:** Association of baseline cognitive reserve index with change in two areas of domain‐specific cognitive performance (memory and processing speed) over a mean follow‐up of 2.71 (SD = 0.73) years.

CRI estimates for	*N*	OR (95% CI)	*p*
Memory	45	2.33 (0.76 to 7.15)	0.138
Processing speed	43	2.01 (0.66 to 6.13)	0.219

*Note*: All analyses adjusted for age, sex, and baseline cognition.

Abbreviations: CI, confidence interval; CRI, cognitive reserve index; OR, odds ratio.

### 3.3 Role of stress measures in association of CRI with cognitive performance

After adjusting Model 1 for the PSS (Table 2), the subjective measure of stress did not alter the significance of associations between CRI and cognitive performance. On adjusting Model 1 for each of the five salivary cortisol measures separately (Table 2), the association of CRI with global cognition was reduced by all except awakening cortisol measure. Adjusting for salivary cortisol measures individually attenuated the associations of CRI with processing speed and working memory. Meanwhile, odds associated with perceptual reasoning were reduced with the lowest and highest reduction observed when adjusted individually for awakening cortisol levels and cortisol AM/PM ratio, respectively. Seven of 30 results had FDR‐adjusted *p* values below 0.05 (Tables S2 and S3) as compared to the FDR‐unadjusted *p* values with 14 of 30 results below the same threshold (Table 2). Differences occurred mainly in reference to associations with perceptual reasoning wherein salivary cortisol measures nullified the association between CRI and perceptual reasoning. On accounting for the use of sleep medication (Table S4), the findings remained similar except for associations of CRI with processing speed and perceptual reasoning when adjusted for PSS and cortisol AM/PM ratio, respectively, wherein the associations were no longer significant.

### 3.4 Modifying role of age, sex, and stress measures between CRI and cognitive performance

There was no evidence that age (*p* for interaction: 0.32 to .87), sex (*p* for interaction: 0.47 to .99), or stress measures (*p* for interaction: 0.11 to .93) modified the association of CRI with domains of cognitive performance except for working memory. Notably, for working memory, an interaction was observed between CRI and cortisol AM/PM ratio (*p* for interaction: 0.09). We examined different thresholds to dichotomize cortisol AM/PM ratio to conduct stratified analysis and found significant differences only at the 20th percentile. For exploratory purpose, we present results by dichotomizing cortisol AM/PM ratio at 2.78. In age and sex‐adjusted analyses stratified by cortisol AM/PM ratio (Table 4), higher CRI was significantly associated with better working memory among individuals with better cortisol AM/PM ratio (range: 3.98 to 42.12, *n* = 60) whereas there was no association among those with lower or unfavorable cortisol AM/PM ratio (range: 0.44 to 2.48, *n* = 18).

**TABLE 4:** Association of cognitive reserve index with working memory stratified by cortisol AM/PM ratio.

CRI estimates for	*N*	Range	Mean (SD)	OR (95% CI)	*p*
Low cortisol AM/PM ratio	18	0.44 to 2.78	1.68 (0.75)	2.12 (0.39 to 11.45)	0.382
High cortisol AM/PM ratio	60	2.93 to 42.12	10.06 (7.82)	4.68 (1.32 to 16.60)	***0.017***

*Note*: All analyses adjusted for age and sex. Low cortisol AM/PM ratio denotes the unfavorable group representing those with ≤20th percentile (ratio ranges from 0.44 to 2.78) of cortisol AM/PM ratio. High cortisol AM/PM ratio representing those with > 20th percentile (ratio ranges from 2.93 to 42.12) of cortisol AM/PM ratio is the favourable group.

Bold italicized is *p* value significant at < 0.05.

Abbreviations: CI, confidence interval; cortisol AM/PM ratio, awakening cortisol/bedtime cortisol; CRI, cognitive reserve index; OR, odds ratio.

### 3.5 Modifying role of cortisol awakening response and age between CRI and AD‐related CSF biomarkers

There was no significant association between CRI and any of the AD‐related CSF biomarkers (Aβ_42_, t‐tau, p‐tau_181_) in the full sample (Table 5). There was evidence of interaction between CRI and the cortisol awakening response in their association with log of p‐tau_181_ (*p* for interaction: 0.08), and between CRI and age in their association with log of p‐tau_181_ (*p* for interaction: 0.01) and log of t‐tau (*p* for interaction: 0.02).

**TABLE 5:** Association of cognitive reserve index with AD‐related CSF biomarkers among participants from memory clinic with subjective cognitive impairment or mild cognitive impairment.

CRI estimates for	*N*	Beta (95% CI)	*p*
Aβ_42_	93	−3.99 (−71.13 to 63.16)	0.906
T‐tau	93	0.04 (−0.10 to 0.17)	0.591
P‐tau_181_	93	0.07 (−0.04 to 0.18)	0.229

*Note*: All analyses adjusted for age and sex.

T‐tau and p‐tau_181_ were log‐transformed for linear regression.

Abbreviations: AD, Alzheimer's disease; Aβ_42_, amyloid beta; CI, confidence interval; CRI, cognitive reserve index; CSF, cerebrospinal fluid; P‐tau_181_, phosphorylated tau.; T‐tau, total tau.

In analysis stratified by tertile of cortisol awakening response (low, medium, and high; *p* for group differences with p‐tau_181 _= 0.035), a higher CRI score was significantly associated with higher levels of log of p‐tau_181_ (beta [β]: 0.27, 95% CI: 0.03 to 0.50; Table S5) among individuals with high cortisol awakening response (*N* = 26), independent of age and sex. This association remained significant even when adjusted for use of sleep medication (β: 0.27, 95% CI: 0.02 to 0.52, *p* = 0.036; data not shown). Given the wide age range of participants (minimum‐maximum: 47.23 to 83.47 years), coupled with significant differences in t‐tau and p‐tau_181_ by age wherein older age groups had higher levels of tau compared to their respective younger counterparts (Table S6), the relation between CRI and tau was explored for multiple age thresholds. Table S7 shows the relation of CRI with the log of t‐tau, and log of p‐tau_181_ stratified by three age cut‐offs (25th, 50th [median], and 75th percentiles). There was a negative linear association between CRI and log of t‐tau (β: −0.30, 95% CI: −0.56 to −0.04) and log of p‐tau_181_ (β: −0.21, 95% CI: −0.41 to −0.0007) among participants aged younger than 56.3 years (Table S7; Young_25_). The direction of association for those 56.3 years (Older_25_) was in the opposite direction but not statistically significant. None of the other age splits showed any association.

## 4 DISCUSSION

Our study, including a composite score of CR and detailed measures of biological (salivary cortisol) and perceived stress in patients from a Swedish memory clinic with available AD biomarkers, highlights four key findings. First, a higher CRI score (protective condition) was associated with better global cognition, particularly in the domains of processing speed, working memory, and perceptual reasoning. Second, diurnal salivary cortisol patterns, which serve as physiological markers of stress, appear to reduce the beneficial influence of high CRI on cognitive performance. Third, there was evidence for interaction between CRI and the cortisol AM/PM ratio in relation to working memory, such that a higher CRI was associated with better working memory in memory clinic patients with a favorable AM/PM ratio alone. Fourth, there was no association between CRI and AD‐related CSF biomarkers in the full analytical sample. However, there was an indication of cortisol awakening ratio and age playing potential modifying roles in the relation between CR and tau pathology.

In line with prior work,^9^, ^55^ this study found higher CR associated with better global and domain‐specific cognitive functioning in individuals with clinically manifest cognitive impairment. CR was linked to higher neurocognitive protection in individuals whose cognitive abilities may have been compromised by accumulating neurodegenerative changes. We did not find an association between CR and episodic/short‐term memory,^56^ although it is possible that CR operates by allowing more flexible strategy usage, an ability believed to be assessed by executive functions tasks, which includes working memory and perceptual reasoning.^57^ This might explain why we saw relationships with all domains but memory. It would be in line with findings from a CR study addressing the impact of sociobehavioral proxies on cognition among cognitively healthy adults.^10^ The study found consistent protective effects of CR on speed/executive trajectories but less consistently on episodic memory.^10^

Our findings highlight, for the first time, the potential modifying role of physiological stress on the CR‐cognition association. Accounting for any of the five measures of salivary cortisol individually attenuated the association of CR with processing speed and working memory. Furthermore, greater CR improved working memory among individuals with higher cortisol AM/PM ratio (ie, favorable condition), but not among those with low cortisol AM/PM ratios. We observed that the relation between CR and working memory varied by the cortisol AM/PM ratio in our cross‐sectional sample of memory clinic participants. It is, however, necessary to replicate these findings with a larger longitudinal sample because it was carried out on a stratified subsample of participants, with a small number of individuals in each category (low and high) of cortisol AM/PM ratio. Contrary to our findings, an earlier cross‐sectional study that examined the interaction between nocturnal salivary cortisol levels and CR but in cognitively healthy older adults did not find any significant modifying effects for neuropsychological performance.^58^ Findings could differ owing to proxy measures (eg, intelligence, number, and fluency of foreign language)^58^ and method (principal component analysis)^58^ utilized to create CR, use of a single salivary cortisol measurement, and varied demographic samples.

Interestingly, adjustment for perceived stress – the subjective measure of psychosocial stress– did not affect the relation of CR with cognitive performance. A study on older adults reported attenuation of the longitudinal relationship between perceived stress and subsequent decline in executive functioning among individuals with high CR over a follow‐up of 6 years.^59^ Results based on individuals with amnestic MCI did not find self‐reported stress parameters measured either by PSS or Recent Life Changes Questionnaire to be associated with adverse cognitive outcomes over a follow‐up of 18 months, unlike with high salivary cortisol levels.^13^ There were weak correlations between the PSS and salivary cortisol measures in our study. It seems that self‐reported psychological stress and the endocrine stress response, that is, salivary cortisol patterns, might indicate distinct constructs rather than being different indicators of the same construct. It was previously noted that there is limited association between cortisol and self‐reported psychological stress with marginal psycho‐endocrine covariance and small proportion of explained variance.^15^ However, theoretical concepts underlying different stress questionnaires vary significantly, and these concepts might be influenced by aspects connected to the evaluation of self‐reported methodologies per se.

We found that among individuals with a high cortisol awakening response, greater CR was associated with higher levels of p‐tau_181_. One potential explanation could be that CR is unable to buffer against AD pathology at abnormally elevated levels of cortisol awakening response among memory clinic patients. It has been shown that the protective role of CR continues into the MCI stage, but this role becomes adverse around the start of the onset of AD.^60^ These memory clinic patients might have reached the threshold for delay in the onset of clinical AD by enhancing CR. Another possible reasoning could be that there is reverse causation between tau protein accumulation and stress. One might argue that tau protein accumulation could predispose an individual to feel more stress and, perhaps, that tau‐related neurodegeneration affects HPA‐axis functioning,^61^ resulting in less control and disengagement from contributors of CR. As we are limited by cross‐sectional design, these hypotheses need to be examined further in longitudinal settings.

There was no evidence of association with change in memory or processing speed over 2.7 years of follow‐up in our sample of memory clinic patients. Findings in adults with subjective cognitive complaints showed a positive effect of CR on cognitive performance at baseline, 18‐ and 36‐months of follow‐up and also showcased the indirect effect of CR on cognitive domains of episodic memory, and overall cognition via working memory domain.^62^ Although another study found that CR influenced cognitive performance at baseline but was not associated with change over 2–3 years among older women.^63^ Direct comparison of the results is challenging due to the variation in methodology, sample population, proxies for CR index construction, and subtests to compose cognitive performance domains.

In our study, CR was unrelated to AD‐related CSF biomarkers. On a similar note, another study based on 91 cognitively normal participants with a median follow‐up of 7 years found no association between reserve score (composite score consisting of education years, occupation level, intelligence quotient, and intracranial volume) and risk of clinical progression to AD/MCI, and the interaction with CSF cortisol levels was also not statistically significant.^64^ In the same study,^64^ a higher CR score was found to be protective on the risk of progression from preclinical stage to AD/MCI among those with high levels of CSF cortisol and abnormal levels of Aβ_42_. Although CR was not associated with AD biomarkers in our study greater CR was related to better cognitive performance, and these associations persisted even after adjusting for Aβ_42_ (data not shown). Together the findings might imply that CR operates by means independent of AD‐pathology among memory clinic patients.

Our study has notable strengths, particularly its novel exploration of multiple biological and psychosocial stress indicators in relation to CR‐cognition‐AD biomarkers in older adults with preclinical/prodromal dementia. This comprehensive approach involved self‐reported perceived stress and physiological stress assessed through five diurnal salivary cortisol measures. The inclusion of saliva‐derived cortisol assessed at multiple time points accounts for sampling differences, whereas using multiple indicators accounts for cortisol circadian rhythm variations. Moreover, non‐invasive salivary samples collected at home mitigates stress responses associated with clinical settings, blood, and CSF sampling.^21^ Our study took into account both early‐life CR and late‐life lifestyle enrichment in memory clinic patients, which provided targeted guidance to improve cognition.

The study's limitations should be noted. First, the study used proxy‐based measures of CR, given that the residual approach comes with important statistical considerations that could further complicate interpretability and limit its usefulness.^65^ Moreover, proxy‐based measures of CR have been shown to rival residual‐based measures in terms of effect on dementia incidence, which underscores the importance of early and midlife factors in preventing dementia in late life.^66^ Second, since large proportion of participants had invalid t3 cortisol data, the cortisol awakening response and daily cortisol output were calculated without t3, which might have diminished their robustness.^49^ Third, AD‐related biomarker measures were unavailable at follow‐up, and no causal relationship with CR could be inferred. Lastly, the relatively small sample size reduced the possibility of drawing a more robust inference from the associations, specifically from the stratified analyses, to examine interactions. But the results of this study can be generalized to clinical populations with characteristics similar to those of our memory clinic cohort.

Our findings suggest that CR might confer neurocognitive benefits for memory clinic patients. It also appears that markers of stress dysregulation may reduce the neurocognitive advantages accumulated via cognitively stimulating and enriching experiences and late‐life lifestyles in individuals with cognitive impairments. The clinical implications of such findings are significant. An expanding body of research, including randomized controlled trials, has suggested that mindfulness and meditation practices have the potential to lower cortisol levels, modulate the cortisol awakening response, alleviate perceived stress, and slow cognitive decline.^67^ Nonetheless, these promising findings necessitate further examination of their potential effectiveness in AD prevention and stress management. Further research is required to explore the underlying mechanisms for the observed associations.

## CONFLICT OF INTEREST STATEMENT

All authors report no conflicts of interest related to the manuscript. Author disclosures are available in the supporting information.

## CONSENT STATEMENT

Research ethics approval for the Co‐STAR study was obtained from the Regional Ethical Review Board (Stockholm) (reference number: 2014/524‐31/1). Only participants whose informed written consent was obtained were included in the data collection.

## Supporting information

Supporting Information

Supporting Information
