# Supplementary material for: Cognitive reserve, cortisol, and Alzheimer's disease biomarkers: A memory clinic study
Source: Alzheimers Dement. 2024 Jun 4;20(7):4486–98. doi: 10.1002/alz.13866 (PMC11247673; doi:10.1002/alz.13866)
Supplement: Supplementary file 1 — Supporting Information [file ALZ-20-4486-s002.docx]

**TABLE OF CONTENTS**

[Supplementary methods: Social health 2](#_Toc155625542)

[Supplementary methods: Leisure activities index 4](#_Toc155625543)

[**Supplementary Figure 1** Construction of social health index 5](#_Toc155625604)

[**Supplementary Figure 2** Construction of leisure activity index 5](#_Toc155625605)

[**Supplementary Table S1** Correlation matrix of perceived stress score and five cortisol measures to assess the diurnal cortisol pattern 6](#_Toc161078926)

[**Supplementary Table S2** Association of cognitive reserve index with global cognition and memory and processing speed domains of cognitive performance among memory clinic participants 7](#_Toc161078927)

[**Supplementary Table S3** Association of cognitive reserve index with working memory and perceptual reasoning domains of cognitive performance among memory clinic participants 8](#_Toc161078928)

[**Supplementary Table S4** Association of cognitive reserve index with cognitive performance among participants from memory clinic with subjective cognitive impairment or mild cognitive impairment when adjusted for stress measures separately with use of sleep medication 9](#_Toc161078929)

[**Supplementary Table S*5*** Association of cognitive reserve index with log of p-tau stratified by cortisol awakening response 10](#_Toc161078930)

[**Supplementary Table S6** Differences in P-tau_181_ and T-tau by age 10](#_Toc161078931)

[**Supplementary Table S7** Association of cognitive reserve index with log of p-tau_181_ and t-tau among memory clinic participants stratified by different age thresholds 11](#_Toc161078932)

# Supplementary methods: Social health

*Marital status*

| **What is your marital status?** | Married/cohabitating partner; window/widower (also after cohabitation); living alone; divorced; living apart |
| --- | --- |

*Frequency of direct or remote contacts*

| **How often do you meet personally with the following:** | |
| --- | --- |
| 1. Parent | For each item:  Daily, more than 2 times per week  Weekly, more than twice a month  Monthly. More than 6 times per year  Quarterly, more than once a year  Less often, rarely  Never |
| 2. Children |  |
| 3. Son-in-law/daughter-in-law |  |
| 4. Grandchildren |  |
| 5. Siblings |  |
| 6. Other relative |  |
| 7. Neighbor |  |
| 8. Friend |  |

| **How often are you in contact with the following via telephone, letters, or email:** | |
| --- | --- |
| 1. Parent | For each item:  Daily, more than 2 times per week  Weekly, more than twice a month  Monthly. More than 6 times per year  Quarterly, more than once a year  Less often, rarely  Never |
| 2. Children |  |
| 3. Son-in-law/daughter-in-law |  |
| 4. Grandchildren |  |
| 5. Siblings |  |
| 6. Other relative |  |
| 7. Neighbor |  |
| 8. Friend |  |

*Social network size*

| How many people do you feel you know well and can talk to about most things? (e.g., relatives, friends, neighbors, and/colleges) | None; 1-2 people; 3 people; 4-6 people; 7-9  people; 10-15 people; 16-30 people; More  than 30 people.  Several options checked |
| --- | --- |

*12 statement questions pertaining to perceived satisfaction with contacts in providing emotional support and instrumental aid:*

| **Questions:** | **Options for each of the items:** |
| --- | --- |
| 1. There is an important person in my life when I need them. 2. There is an important person in my life with whom I can share joy and sorrow. 3. My family is really trying to help me. 4. I receive the emotional help and support from my family that I need. 5. There is one person who is important to me who really contributes to my well-being. 6. My friends try really hard to help me. 7. If something goes wrong, I can count on help from my friends. 8. I can talk to my family about my problems. 9. I have friends with whom I can share joy and sorrow. 10. There is an important person in my life who cares about my feelings. 11. My family is willing to support me in my decisions. 12. I can talk about my problems with my friends. | Strongly disagree; very low level of agreement; disagree to a low degree; partially agree; strongly agree; agree to very high degree; strongly agree. |

# Supplementary methods: Leisure activities index

#

| Have you participated in any of the following entertainment or cultural activities in the last year? | |
| --- | --- |
| 1. Cinema/Theatre/Concert 2. Sporting Events 3. Museum/Art exhibit 4. Go to Restaurant/Pub/Cafe 5. Bingo 6. Dancing 7. Attended church or a revival meeting 8. Participated in a study circle or a course 9. Involved in non-profit activities 10. Participated in association work 11. Travelling | For each item select one option:  Every week  Every month  Less frequently  Never |

| Have you engaged in any of the following productive leisure activities in the last year? | |
| --- | --- |
| 1. Gardening 2. Hiking in forest/picking berries, mushrooms 3. Hunting, fishing 4. Knitting, weaving, sewing 5. Painting, drawing, working with clay/pottery 6. Carrying out home repairs 7. Car or mechanical repairs | For each item select one option:  Every week  Every month  Less frequently  Never |

| Have you engaged in any of the following other activities in the last year? | |
| --- | --- |
| 1. Follow news, on internet, newspaper 2. Read book 3. Watch TV 4. Play chess/card games 5. Solve crosswords or sudoku 6. Play musical instrument 7. Listen to music 8. Use the internet or play computer games | For each item select one option:  Every week  Every month  Less frequently  Never |

| Do you exercise regularly with light exercise? (e.g., walking on roads and in parks, forest walks, short bike rides, light exercise, golf) | Never; less frequently; 2 to 3 times per month; several times per week; everyday |
| --- | --- |

| Do you exercise regularly with more intense exercise? (e.g., jogging, brisk long walks, heavy gardening, long bike rides, intensive gymnastics, long distance skating, skiing, swimming, playing ball (not golf) or other similar activities) | Never; less frequently;2 2 to 3 times per month; several times per week; everyday |
| --- | --- |

***
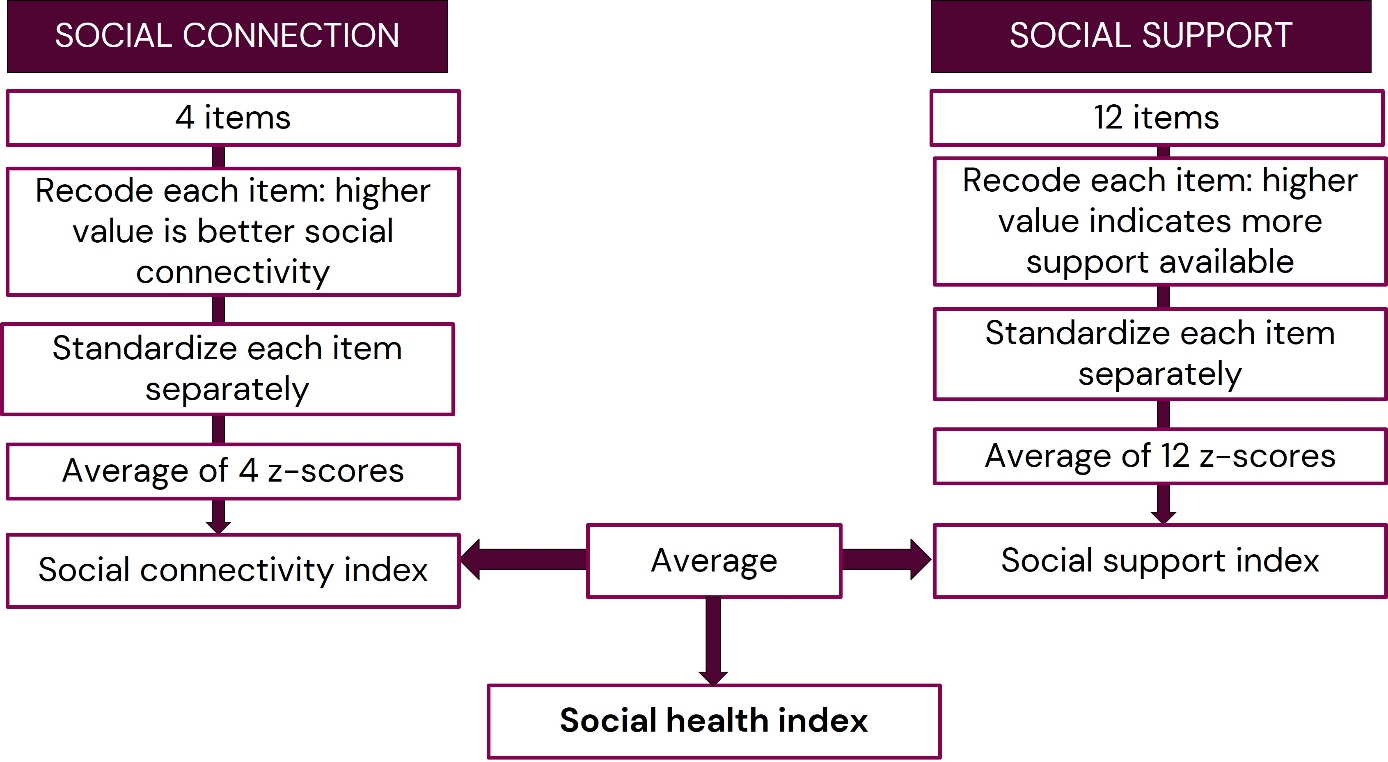
***

**Supplementary Figure 1** Construction of social health index

***
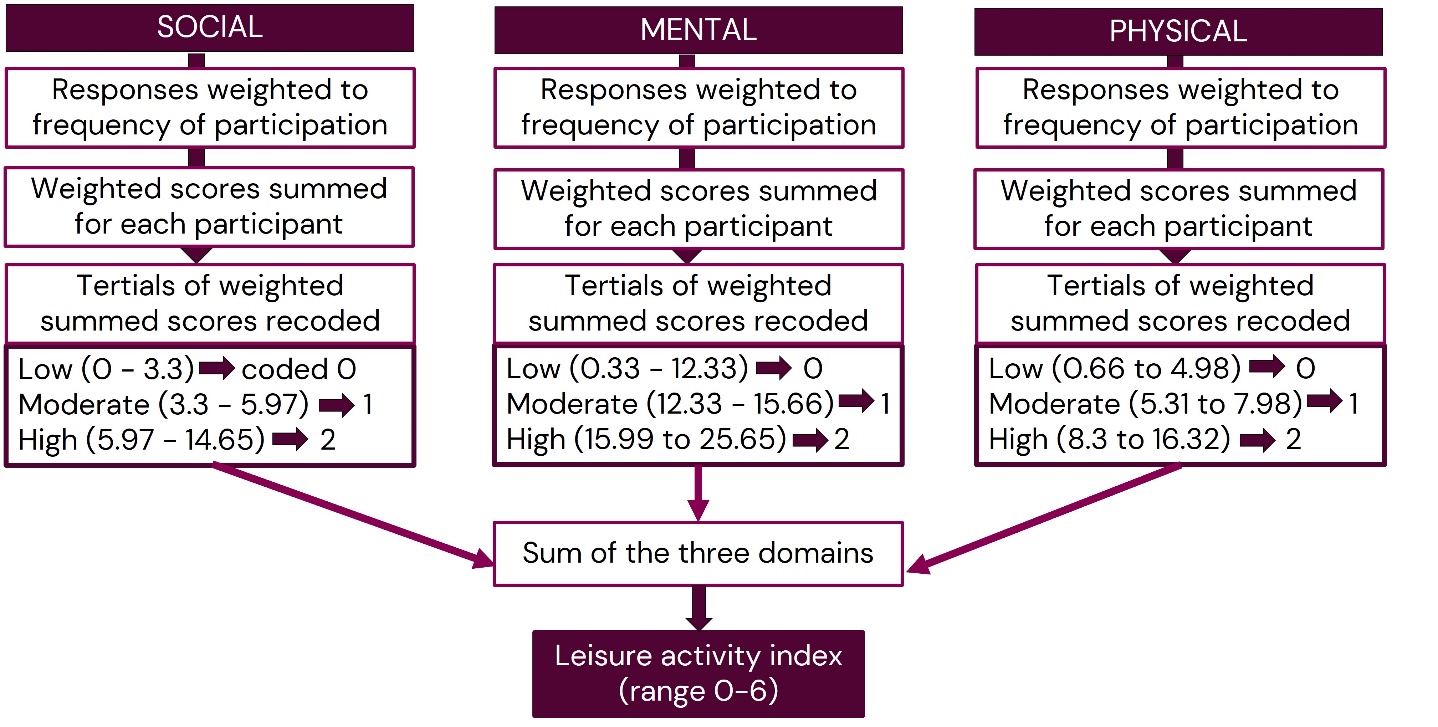
***

**Supplementary Figure 2** Construction of leisure activity index

**Supplementary Table S1** Correlation matrix of perceived stress score and five cortisol measures to assess the diurnal cortisol pattern

|  | **[1]** | **[2]** | **[3]** | **[4]** | **[5]** | **[6]** |
| --- | --- | --- | --- | --- | --- | --- |
| Perceived stress score [1] | 1.00 |  |  |  |  |  |
| Awakening cortisol levels [2] | -0.02 | 1.00 |  |  |  |  |
| Bedtime cortisol levels [3] | 0.19 | 0.44 | 1.00 |  |  |  |
| Cortisol awakening response [4] | -0.13 | -0.32 | 0.03 | 1.00 |  |  |
| Total daily cortisol output [5] | 0.01 | 0.47 | 0.68 | 0.26 | 1.00 |  |
| Ratio of awakening to bedtime cortisol levels [6] | -0.21 | 0.14 | -0.82 | -0.26 | -0.43 | 1.00 |

**Supplementary Table S2** Association of cognitive reserve index with global cognition and memory and processing speed domains of cognitive performance among memory clinic participants

| **CRI score** | **Global cognition**  **(N = 91)** | | | **Memory**  **(N = 98)** | | | **Processing speed**  **(N = 92)** | | |
| --- | --- | --- | --- | --- | --- | --- | --- | --- | --- |
|  | **OR (95% CI)** | **P** | **P (FDR)^1^** | **OR (95% CI)** | **P** | **P (FDR)^1^** | **OR (95% CI)** | **P** | **P (FDR)^1^** |
| **Adjustment for stress measures** | |  |  |  |  |  |  |  |  |
| **Perceived stress scale** | 6.86 (2.11-22.32) | ***0.001*** | ***0.024*** | 1.88 (0.82-4.32) | 0.139 | 0.16 | 2.52 (1.02-6.28) | ***0.046*** | 0.084 |
| **Awakening cortisol** | 5.27 (1.74-15.91) | ***0.003*** | ***0.024*** | 1.69 (0.73-3.93) | 0.221 | 0.24 | 2.34 (0.91-5.97) | 0.077 | 0.100 |
| **Bedtime cortisol** | 4.81 (1.59-14.49) | ***0.005*** | ***0.025*** | 1.60 (0.68-3.74) | 0.278 | 0.29 | 2.50 (0.98-6.35) | 0.055 | 0.084 |
| **CAR** | 5.09 (1.68-15.40) | ***0.004*** | ***0.024*** | 1.95 (0.81-4.71) | 0.139 | 0.16 | 2.46 (0.97-6.24) | 0.059 | 0.084 |
| **Daily cortisol output** | 5.07 (1.68-15.33) | ***0.004*** | ***0.024*** | 1.72 (0.74-4.01) | 0.207 | 0.23 | 2.44 (0.97-6.14) | 0.058 | 0.084 |
| **Cortisol AM/PM ratio** | 4.49 (1.46-13.78) | ***0.009*** | ***0.039*** | 1.57 (0.66-3.73) | 0.307 | 0.31 | 2.29 (0.88-5.98) | 0.091 | 0.110 |

^1^False discovery rate (FDR)-adjusted p-value using the Simes-Benjamini-Hochberg method implemented using the Stata qqvalue package.

***Bold Italicized*** is p value significant at <0.05.

In addition to adjustment for each of the six different stress measures in separate analytical models, all analyses are adjusted for age and sex.

Abbreviations: cortisol AM/PM ratio, awakening cortisol/bedtime cortisol; CRI, cognitive reserve index; OR, odds ratio; CI, confidence interval; CAR, cortisol awakening response; FDR, false discovery rate.

**Supplementary Table S3** Association of cognitive reserve index with working memory and perceptual reasoning domains of cognitive performance among memory clinic participants

| **CRI score** | **Working memory**  **(N = 78)** | | | **Perceptual reasoning**  **(N = 93)** | | |
| --- | --- | --- | --- | --- | --- | --- |
|  | **OR (95% CI)** | **P** | **P (FDR)^1^** | **OR (95% CI)** | **P** | **P (FDR)^1^** |
| **Adjustment for stress measures** | |  |  |  |  |  |
| **Perceived stress scale** | 3.70 (1.27-10.78) | ***0.016*** | 0.060 | 4.52 (1.72-1.89) | ***0.002*** | ***0.024*** |
| **Awakening cortisol** | 2.72 (0.98-7.56) | 0.055 | 0.084 | 2.96 (1.19-7.34) | ***0.019*** | 0.063 |
| **Bedtime cortisol** | 2.58 (0.93-7.14) | 0.068 | 0.093 | 2.68 (1.07-6.68) | ***0.035*** | 0.084 |
| **CAR** | 2.70 (0.98-7.45) | 0.056 | 0.084 | 2.84 (1.15-7.02) | ***0.024*** | 0.068 |
| **Daily cortisol output** | 2.71 (0.98-7.53) | 0.055 | 0.084 | 2.82 (1.14-6.99) | ***0.025*** | 0.068 |
| **Cortisol AM/PM ratio** | 2.80 (0.98-7.99) | 0.054 | 0.084 | 2.60 (1.03-6.57) | ***0.043*** | 0.084 |

^1^False discovery rate (FDR)-adjusted p-value using the Simes-Benjamini-Hochberg method implemented using the Stata qqvalue package.

***Bold Italicized*** is p value significant at <0.05.

In addition to adjustment for each of the six different stress measures in separate analytical models, all analyses are adjusted for age and sex.

Abbreviations: cortisol AM/PM ratio, awakening cortisol/bedtime cortisol; CRI, cognitive reserve index; OR, odds ratio; CI, confidence interval; CAR, cortisol awakening response; FDR, false discovery rate.

**Supplementary Table S4** Association of cognitive reserve index with cognitive performance among participants from memory clinic with subjective cognitive impairment or mild cognitive impairment when adjusted for stress measures separately with use of sleep medication

| **CRI score** | **Global cognition** | | **Memory** | | **Processing speed** | | **Working memory** | | **Perceptual reasoning** | |
| --- | --- | --- | --- | --- | --- | --- | --- | --- | --- | --- |
|  | **OR (95% CI)** | **P** | **OR (95% CI)** | **P** | **OR (95% CI)** | **P** | **OR (95% CI)** | **P** | **OR (95% CI)** | **P** |
| **Adjustment for stress measures** | | | | | | | | | |  |
| **Perceived stress scale** | 6.96 (2.11-23.02) | ***0.001*** | 1.86 (0.81-4.27) | 0.144 | 2.47 (1.00-6.14) | 0.051 | 3.73 (1.26-11.05) | ***0.018*** | 4.55 (1.72-12.04) | ***0.002*** |
| **Awakening cortisol** | 5.36 (1.74-16.49) | ***0.003*** | 1.67 (0.72-3.87) | 0.234 | 2.30 (0.90-5.88) | 0.082 | 2.69 (0.95-7.62) | 0.062 | 2.94 (1.18-7.32) | ***0.021*** |
| **Bedtime cortisol** | 4.91 (1.59-15.13) | ***0.006*** | 1.56 (0.67-3.65) | 0.303 | 2.43 (0.96-6.19) | 0.062 | 2.51 (0.90-7.02) | 0.080 | 2.63 (1.05-6.61) | ***0.040*** |
| **CAR** | 5.21 (1.69-16.11) | ***0.004*** | 1.92 (0.80-4.64) | 0.146 | 2.39 (0.94-6.08) | 0.067 | 2.68 (0.95-7.54) | 0.061 | 2.83 (1.14-7.03) | ***0.025*** |
| **Daily cortisol output** | 5.25 (1.68-16.43) | ***0.004*** | 1.70 (0.73-3.94) | 0.220 | 2.39 (0.95-6.01) | 0.063 | 2.70 (0.95-7.65) | 0.062 | 2.80 (1.12-6.98) | ***0.027*** |
| **Cortisol AM/PM ratio** | 4.59 (1.46-14.45) | ***0.009*** | 1.53 (0.64-3.63) | 0.337 | 2.21 (0.84-5.78) | 0.106 | 2.67 (0.93-7.68) | 0.068 | 2.54 (1.00-6.49) | 0.051 |

***Bold Italicized*** is p value significant at <0.05.

All analyses are adjusted for age, sex, and use of sleep medication in addition to adjustment for each of the six different stress measures in separate analytical models.

Abbreviations: cortisol AM/PM ratio, awakening cortisol/bedtime cortisol; CRI, cognitive reserve index; OR, odds ratio; CI, confidence interval; CAR, cortisol awakening response.

**Supplementary Table S*5*** Association of cognitive reserve index with log of p-tau stratified by cortisol awakening response

| **CRI estimates for log of P-tau_181_** | **N** | **Range** | **Mean (SD)** | **Beta (95% CI)** | **P** |
| --- | --- | --- | --- | --- | --- |
| Low/blunted CAR | 29 | -2.24 - 0.16 | -0.57 (0.62) | 0.00 (-0.20 - 0.20) | 0.992 |
| Medium CAR | 28 | 0.17 - 1.07 | 0.65 (0.29) | -0.16 (-0.34 - 0.02) | 0.072 |
| High CAR | 26 | 1.08 - 4.64 | 2.09 (0.93) | 0.27 (0.03 - 0.50) | 0.026 |

All analyses adjusted for age and sex. P-tau_181_ was log-transformed for linear regression.

Abbreviations: CRI, cognitive reserve index; P-tau_181_, phosphorylated tau; SD, standard deviation; CI, confidence interval; CAR, cortisol awakening response.

**Supplementary Table S6** Differences in P-tau_181_ and T-tau by age

|  |  |  | **T-tau** | | **P-tau** | |
| --- | --- | --- | --- | --- | --- | --- |
|  | **Age range (years)** | **N** | **Mean (SD)** | **P value** | **Mean (SD)** | **P value** |
| **25^th^ percentile** | | | | | | |
| **Young_25_** | 47.23 to 56.30 | 23 | 250.52 (99.71) | 0.230 | 38.10 (12.08) | 0.053 |
| **Older_25_** | 56.36 to 83.47 | 70 | 335.26 (166.15) |  | 46.59 (19.57) |  |
| **50^th^ percentile** | | | | | | |
| **Young_50_** | 47.23 to 61.04 | 46 | 252.52 (95.01) | ***<0.001*** | 38.07 (11.84) | **<0.001** |
| **Older_50_** | 61.39 to 83.47 | 47 | 374.77 (180.22) |  | 50.77 (21.28) |  |
| **75^th^ percentile** | | | | | | |
| **Young_75_** | 47.23 to 64.77 | 69 | 277 (126.36) | ***<0.001*** | 40.43 (14.95) | ***<0.001*** |
| **Older_75_** | 64.93 to 83.47 | 24 | 421.54 (185.32) |  | 56.13 (22.17) |  |

***Bold*** ***Italicized*** is p value significant at <0.05.

**Supplementary Table S7** Association of cognitive reserve index with log of p-tau_181_ and t-tau among memory clinic participants stratified by different age thresholds

| **25^TH^ PERCENTILE SPLIT** | | | | | | | |
| --- | --- | --- | --- | --- | --- | --- | --- |
| **Young_25_*:* mean [SD] = 53.27 (2.43) years; range 47.23 to 56.3 years** | | | |  | **Older_25_*:* mean [SD] = 64.42 (6.00); range 56.36 to 83.47 years** | | |
| **Outcome** | **N** | **Beta (95% CI)** | **P value** |  | **N** | **Beta (95% CI)** | **P value** |
| T-Tau | 23 | -0.30 (-0.56 - -0.04) | ***0.026*** |  | 70 | 0.11 (-0.06 - 0.28) | 0.195 |
| P-Tau | 23 | -0.21 (-0.41 - -0.0007) | ***0.049*** |  | 70 | 0.13 (-0.01 - 0.27) | 0.063 |
| **50^TH^ PERCENTILE SPLIT [median split]** | | | | | | | |
| **Young_50_*:* mean [SD] = 56.09 (3.46) years; range 47.23 to 61.04 years** | | | |  | **Older_50_*:* mean [SD] = 67.12 (5.52); range 61.39 to 83.47 years** | | |
| **Outcome** | **N** | **Beta (95% CI)** | **P value** |  | **N** | **Beta (95% CI)** | **P value** |
| T-Tau | 46 | -0.06 (-0.23 - 0.12) | 0.511 |  | 47 | 0.06 (-0.16 - 0.28) | 0.586 |
| P-Tau | 46 | -0.02 (-0.16 - 0.12) | 0.827 |  | 47 | 0.10 (-0.09 - 0.28) | 0.289 |
| **75^TH^ PERCENTILE SPLIT** | | | | | | | |
| **Young_75_*:* mean [SD] = 58.42 (4.41) years; range 47.23 to 64.77 years** | | | |  | **Older_75_*:* mean [SD] = 70.99 (5.24); range 64.93 to 83.47 years** | | |
| **Outcome** | **N** | **Beta (95% CI)** | **P value** |  | **N** | **Beta (95% CI)** | **P value** |
| T-Tau | 69 | 0.04 (-0.12 - 0.20) | 0.615 |  | 24 | 0.06 (-0.23 - 0.35) | 0.686 |
| P-Tau | 69 | 0.05 (-0.08 - 0.18) | 0.426 |  | 24 | 0.13 (-0.13 - 0.39) | 0.302 |

***Bold*** ***Italicized*** is p value significant at <0.05.

All analyses adjusted for sex.

P-tau_181_ and T-tau were log-transformed for linear regression.

Abbreviations: CRI, cognitive reserve index; CI, confidence interval; Aβ_42_, beta-amyloid; T-tau, total tau; P-tau_181_, phosphorylated tau.
